# A novel inhibitor of the insulin/IGF signaling pathway protects from age-onset, neurodegeneration-linked proteotoxicity

**DOI:** 10.1111/acel.12171

**Published:** 2013-11-22

**Authors:** Tayir El-Ami, Lorna Moll, Filipa Carvalhal Marques, Yuli Volovik, Hadas Reuveni, Ehud Cohen

**Affiliations:** 1 Department of Biochemistry and Molecular Biology, The Institute for Medical Research Israel – Canada (IMRIC), The Hebrew University School of MedicineJerusalem, Israel; 2 Faculty of Medicine, Center of Ophthalmology and Vision Sciences (COCV), Institute for Biomedical Research in Light and Image (IBILI), University of CoimbraCoimbra, Portugal; 3 NovoTyr Therapeutics Ltd.Tel Hai, Israel

**Keywords:** aging, *C. elegans*, insulin/IGF signaling inhibitor, neurodegeneration, proteostasis

## Abstract

Aging manipulation is an emerging strategy aimed to postpone the manifestation of late-onset neurodegenerative disorders such as Alzheimer’s (AD) and Huntington’s diseases (HD) and to slow their progression once emerged. Reducing the activity of the insulin/IGF signaling cascade (IIS), a prominent aging-regulating pathway, protects worms from proteotoxicity of various aggregative proteins, including the AD-associated peptide, Aβ- and the HD-linked peptide, polyQ40. Similarly, IGF1 signaling reduction protects mice from AD-like disease. These discoveries suggest that IIS inhibitors can serve as new drugs for the treatment of neurodegenerative maladies including AD and HD. Here, we report that NT219, a novel IIS inhibitor, mediates a long-lasting, highly efficient inhibition of this signaling cascade by a dual mechanism; it reduces the autophosphorylation of the IGF1 receptor and directs the insulin receptor substrates 1 and 2 (IRS 1/2) for degradation. NT219 treatment promotes stress resistance and protects nematodes from AD- and HD-associated proteotoxicity without affecting lifespan. Our discoveries strengthen the theme that IIS inhibition has a therapeutic potential as a cure for neurodegenerative maladies and point at NT219 as a promising compound for the treatment of these disorders through a selective manipulation of aging.

## Introduction

Aberrant protein aggregation is mechanistically linked to the emergence of late-onset human neurodegenerative disorders such as Parkinson’s, Alzheimer’s (AD) (Selkoe, 2003) and Huntington’s (HD) (Bates, 2003) diseases. Although the nature of the aggregating proteins and the mechanisms that underlie the development of these maladies differ greatly, they share similar temporal emergence patterns, while familial, mutation-linked cases onset during the fifth or sixth decade of life, sporadic cases do not manifest earlier than the seventh decade (Amaducci & Tesco, 1994) (HD solely appears as a familial disorder). This common feature defines aging as the major risk factor for the development of these disorders and suggests that aging enables their emergence late in life. During the last decade, this idea has been supported by a plethora of studies (Morley *et al*., 2002; Hsu *et al*., 2003; Cohen *et al*., 2006), which raised the prospect that aging-manipulating drugs could harness the mechanisms that protect the young organism from disease to postpone the onset of neurodegeneration and delay its progression (Morimoto, 2006).

Reducing the activity of the insulin/IGF signaling cascade (IIS), a highly conserved aging-regulating pathway, elevates stress resistance and extends lifespans of worms, flies (Kenyon, 2005) and mice (Holzenberger *et al*., 2003). In the nematode *Caenorhabditis elegans* (*C. elegans*), the tyrosine kinase DAF-2 is the sole IIS receptor (Kimura *et al*., 1997). Upon activation, DAF-2 initiates a signaling cascade that negatively regulates the activity of at least three transcription factors: DAF-16/FOXO (Lee *et al*., 2001), SKN-1/NRF (Tullet *et al*., 2008) and the heat shock factor 1 (HSF-1) (Chiang *et al*., 2012). The IIS mitigates the activity of DAF-16/FOXO and SKN-1/NRF by activating its downstream kinases, AKT-1 and PDK-1 (Paradis & Ruvkun, 1998), which phosphorylate these transcription factors, preventing them from entering the nucleus and from regulating their target genes. Similarly, the IIS negatively regulates HSF-1 by preventing the phosphorylation of DDL-1, an HSF-1-interacting protein that upon phosphorylation detaches from HSF-1, enabling its entry to the nucleus (Chiang *et al*., 2012). Thus, IIS reduction hyperactivates its downstream transcription factors creating youthful, long-lived, stress resistant worms (Kenyon, 2005).

The mammalian signaling pathway downstream of the insulin-like growth factor 1 (IGF1) is similar to the worm’s IIS. Upon IGF1 binding, the IGF1 receptor (IGF1R) undergoes autophosphorylation, followed by the recruitment and phosphorylation of the insulin receptor substrates (IRS) 1 and 2 on tyrosine residues. These events lead to the phosphorylation and activation of AKT that result in the phosphorylation of the forkhead box class O (FOXO) family of transcription factors. Phosphorylated FOXO molecules are prevented from entering the nucleus and from regulating their target genes [reviewed in (Partridge & Bruning, 2008)]. Reduced IGF1 signaling by the deletion of one copy of *Igf1r* (that encodes the IGF1R), an orthologue of *daf-2*, extends lifespan and elevates oxidative stress resistance of mice (Holzenberger *et al*., 2003). Analogously, mutations in IIS components are associated with extreme longevity of humans of different ethnicities (Suh *et al*., 2008; Barbieri *et al*., 2010), suggesting that the longevity mechanism downstream of the IIS is conserved from worms to humans.

To test whether slowing aging by IIS reduction protects from toxic protein aggregation (proteotoxicity), we utilized worms that express the highly aggregative, human AD-associated peptide, Aβ_3-42_, in their body wall muscles (strain CL2006, Aβ worms) (Link, 1995). The expression of Aβ in these animals leads to a progressive paralysis within the worm population. We discovered that reducing the IIS by *daf-2* RNA interference (RNAi) alleviates Aβ-associated paralysis. This protection was conferred by opposing activities: HSF-1 regulates disaggregation while DAF-16 mediates hyperaggregation to create high molecular weight Aβ aggregates of lower toxicity (Cohen *et al*., 2006). The counter-proteotoxic effect of IIS reduction is associated with Aβ hyperaggregation (Cohen *et al*., 2006) and temporally separable from longevity (Cohen *et al*., 2010).

IIS reduction was also reported to protect model nematodes from proteotoxicity of other neurodegeneration-linked, aggregative proteins including HD-associated polyQ stretches (Morley *et al*., 2002) and the amyotrophic lateral sclerosis (ALS)-linked mutated protein, TAR DNA binding protein 43 (TDP-43) (Zhang *et al*., 2011).

Analogously to worms, mice that harbor only one *Igf1r* copy are protected from behavioral and pathological impairments that stem from the aggregation of human Aβ in the brain. This protection is promoted by the formation of densely packed Aβ fibrils (Cohen *et al*., 2009).

Collectively, these studies point at IIS reduction as a promising strategy toward the development of novel neurodegeneration therapies (Morimoto, 2006) and call for the assessment of IGF1 signaling inhibitors as novel drugs for the treatment of neurodegenerative disorders (Cohen, 2011).

We have recently developed a novel family of IGF1 signaling inhibitors (the NT compounds) that target IRS1/2 for degradation and facilitate a long-lasting inhibitory effect (Reuveni *et al*., 2013). Here, we show that NT219, a new member of the NT family, reduces the IGF1R kinase activity and targets IRS1/2 to elimination, and thus effectively inhibits IGF1 signaling in human cells. In *C. elegans*, NT219 reduces IIS activity, activates protective gene networks, elevates stress resistance and protects from Aβ- and polyQ-mediated proteotoxicity, without affecting lifespan. Our findings suggest that NT219 may be a novel therapy for Alzheimer’s disease and other human neurodegenerative maladies.

## Results

### NT219 effectively inhibits the IGF1 signaling pathway in human cells

**Figure 1:**
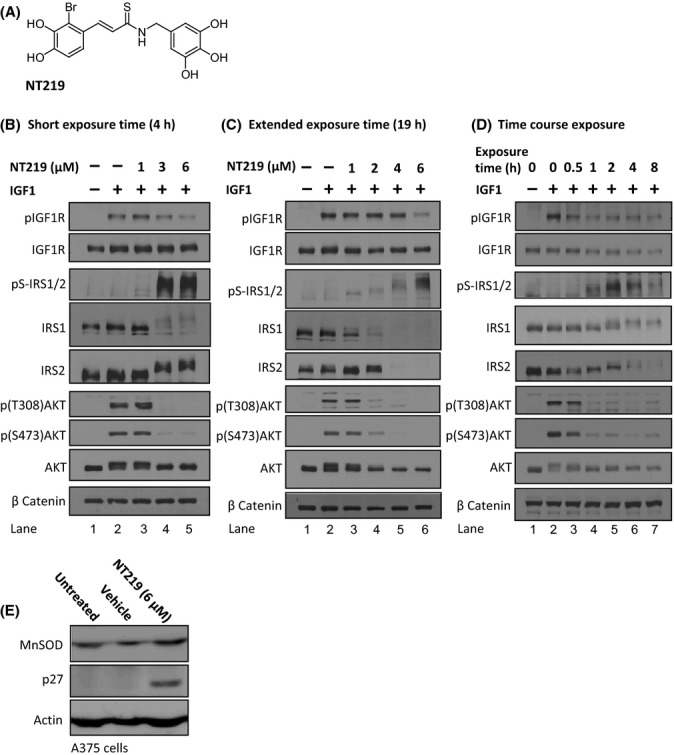
NT219 inhibits the IGF1R to AKT signaling pathway in human melanoma cells. (A) Chemical formula of NT219. (B–C) NT219 treatment reduces the IGF-induced autophosphorylation of the IGF1R, induces inhibitory Ser-phosphorylation and subsequent elimination of the insulin receptor substrate 1 (IRS1) and 2 (IRS2), and prevents the IGF1-induced activation of AKT in A375 human melanoma cells, following short (B) and long (C) exposures. (D) Temporal analysis of the effects of NT219 on components of the IGF pathway indicates that the compound acts on the IGF1R and AKT within 1 h to block the signaling cascade and confers Ser-phosphorylation and degradation of IRS1 and IRS2 2–8 h after exposure to achieve long-term inhibition. (E) NT219 elevates the expression levels of the FOXO target genes p27 and manganese superoxide dismutase (MnSOD) in A375 cells.

To assess the efficiency of NT219 (Fig. 1A) as an inhibitor of IGF1 signaling and to explore its mechanism of action, we used the human melanoma cell line, A375. The cells were treated with increasing NT219 concentrations for either 4 h (Fig. 1B) or 19 h (Fig. 1C), and the IGF1 signaling cascade was activated by the supplementation of IGF1 to the cell media. Western blot (WB) analysis showed that IGF1 induced the autophosphorylation of IGF1R (pIGF1R, Fig. 1B,C, lane 2), and that this phosphorylation was largely abolished after treatment with 6 μm and that this phosphorylation was largely abolished after treatment with 6mM NT219 (pIGF1R, Fig. 1B lane 5 and 1C lane 6). Since upon activation IGF1R undergoes auto-phosphorylation (Favelyukis *et al*., 2001) this observation indicates that NT219 reduces the activation of IGF1R.

The phosphorylation of IRS1 and IRS2 on serine residues leads to their degradation. This negative feedback loop enables cells to shut off IGF1 signaling (Boura-Halfon & Zick, 2009). NT219-related compounds uniquely induce Ser-phosphorylation and degradation of IRS1 and IRS2 to gain a long-lasting inhibition of IGF1 signaling (Reuveni *et al*., 2013). Using WB and specific antibodies, we found that both IRS1 and IRS2 undergo phosphorylation on serine residues after a short exposure to NT219 (pS-IRS1/2, Fig. 1B lanes 4–5) followed by their elimination (IRS1 and IRS2, Fig. 1C, lanes 4–6).

To further scrutinize the effect of NT219 on IGF1 signaling, we examined the rate of phosphorylation of the kinase AKT, a downstream target of the pathway whose phosphorylation is a prerequisite for its activation in various tissues (Bondy & Cheng, 2004). NT219 treatment abolished the IGF1-induced activation of AKT after short and long exposures, demonstrated by the inhibition of AKT phosphorylation on threonine 308 and serine 473 (Fig. 1B,C, lanes 4–6).

To explore the temporal order of events after treatment with NT219, we performed a kinetic experiment in which the cells were treated with the compound for the indicated times (Fig. 1D, 0.5–8 h), stimulated with IGF1 for 5 min, and subjected to WB. We found that partial inhibition of IGF1R autophosphorylation is achieved merely half an hour after the addition of the drug, leading to reduced AKT phosphorylation (Fig. 1D). Long-term inhibition of AKT activation is attained when the second inhibitory mechanism, involving Ser-phosphorylation and degradation of IRS1 and IRS2, occurs (Fig. 1D lanes 4–7). These results show that phosphorylation precedes the degradation of IRS1 and IRS2.

Finally, we asked whether NT219 treatment elevates the level of proteins that are encoded by FOXO target genes. A375 cells were treated for 19 h with 6 μm NT219 or the vehicle, and the levels of manganese superoxide dismutase (MnSOD) and of the cell cycle regulator p27, both encoded by FOXO-regulated genes (van der Vos & Coffer, 2011), were tested by WB. Our results (Fig. 1E) reveal increased levels of both proteins in NT219-treated cells compared with untreated and vehicle-exposed cells [NT219 also increased the levels of MnSOD in neuroblastoma 2a (N2a) cells (Fig. S1)].

Together, our results show that NT219 is an efficient IGF1 signaling inhibitor which acts by a dual-step mechanism to elevate the transcription of the pathway’s target genes.

### Elevated expression of IIS-regulated genes in NT219-treated worms

**Figure 2:**
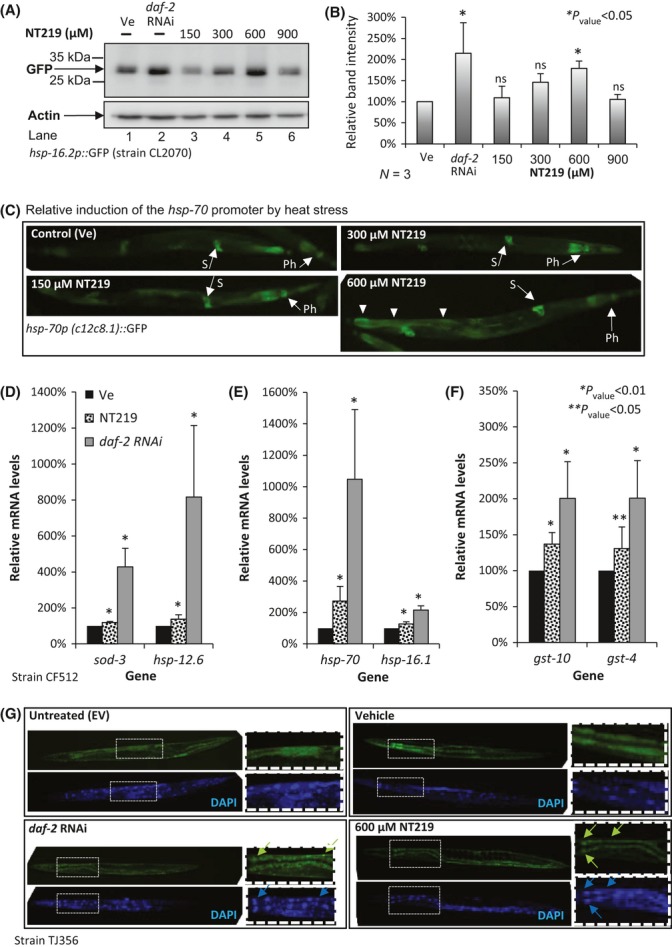
600 μm NT219 efficiently induces the expression of IIS target genes in *C. elegans*. (A) Worms expressing GFP under the regulation of the *hsp-16.2* promoter (strain CL2070) were treated for 3 h at days 1 and 2 of adulthood with the indicated NT219 concentrations. Western blot analysis shows that NT219 activates the *hsp-16.2* promoter in a dose-dependent manner up to 600 μm. Treatment with 900 μm NT219 exhibited lower activation effect compared with the level seen after treatment with 600 μm. (B) Three independent experiments confirmed the significance of the results presented in (A). (C) 600 μm NT219, but not lower concentrations, elevates the expression level of GFP driven by the *hsp-70* promoter after exposure to heat (33°C, 3 h) as visualized by fluorescent microscopy. This effect was foremost prominent in the intestine (arrowheads) (‘Ph’ – pharynx, ‘S’ – spermatheca). (D–F) Relative expression levels of DAF-16 (D), HSF-1 (E), and SKN-1 (F) target genes in vehicle-treated (black bars), NT219-treated (dashed bars), and *daf-2* RNAi-treated CF512 worms as measured by real-time quantitative PCR. Bars represent average values of three independent experiments ± SEM. (G) DAF-16::GFP chimera enters the nuclei of NT219-treated worms, but not of their vehicle-treated counterparts.

In worms, IIS reduction elevates the induction rate of the HSF-1 target gene *hsp-16.2* upon exposure to heat beyond the induction level of untreated animals (Hsu *et al*., 2003; McColl *et al*., 2010). We exploited this feature to assess whether NT219 is capable of reducing IIS in *C. elegans* by employing worms that express the green fluorescent protein (GFP) under the regulation of the *hsp-16.2* promoter (strain CL2070). CL2070 worms were grown on control bacteria harboring the empty vector (EV). The animals were then divided to identical groups and treated at days 1 and 2 of adulthood for 3 h with the chemical vehicle of NT219 [Fig. 2A, lane 1 (Ve)], or with 150, 300, 600 or 900 μm NT219 (lanes 3–6, respectively). An identical worm group was grown from hatching on *daf-2* RNAi bacteria (lane 2). At day 2 of adulthood, all worm groups were exposed to 33°C for 3 h and harvested, and GFP levels were analyzed by WB. We found that NT219 increased the *hsp-16.2* induction levels in heat-stressed animals in a dose-dependent manner. While treatment with 150 μm NT219 had no effect on the level of GFP, 300 μm had a moderate effect and 600 μm NT219 remarkably increased the level of GFP to be similar to that of *daf-2* RNAi-treated worms. Treatment with 900 μm NT219 had lower effect than that of 600 μm of the drug. Three independent repeats confirmed these observations (Fig. 2B and S2).

To compare the efficiencies of NT219 and of BMS-754807, a kinase inhibitor of IGF1R (Carboni *et al*., 2009), as activators of the *hsp-16.2* promoter, we exploited CL2070 worms. The animals were treated with 150–900 μm BMS-754807, and GFP levels were examined by WB. Our results showed no elevation in GFP levels in BMS-754807-treated animals (Fig. S3). This observation suggests that in the worm, NT219 is a more efficient IIS inhibitor than the other compound.

We also tested whether NT219 affects the induction of the HSF-1 target gene, *hsp-70* by treating worms that express GFP under the control of the *hsp-70* promoter (*hsp-70p*::GFP) with the vehicle (Ve), 150, 300 or 600 μm NT219, exposing them to heat (33°C, 3 h) and visualizing them. An increase in GFP signal was observed in worms after treatment with 600 μm NT219, but not after treatment with lower concentrations (Fig. 2C, 600 μm NT219 arrowheads).

Our results showed that 600 μm NT219 most efficiently inhibits the IIS and elevates the expression of its downstream genes upon exposure to heat. Thus, we used this NT219 concentration for all worm-based experiments described below.

Using quantitative real-time PCR (qPCR) and specific primer sets, we tested the effect of NT219 on the expression of DAF-16, HSF-1, and SKN-1 target genes in temperature sensitive sterile worms (strain CF512). These nematodes lack progeny when exposed to 25°C during development [this mild heat stress does not affect the expression of heat shock proteins (Volovik *et al*., 2012)], a feature that enabled us to examine the expression levels of IIS target genes in adult worms with no background of gene expression in embryos. The worms were grown on EV bacteria and treated for 3 h with 600 μm NT219 at days 1 and 2 of adulthood. An identical group of worms was treated with the vehicle (Ve), and a third group was grown on *daf-2* RNAi bacteria. For the qPCR reactions, we used primers toward the DAF-16 target genes *sod-3* and *hsp-12.6* (Murphy *et al*., 2003), the HSF-1 regulated genes *hsp-16.1* (Link *et al*., 1999) and *hsp-70,* and the SKN-1 controlled genes *gst-4* and *gst-10* (Wang *et al*., 2010). Three independent experiments showed that NT219 (Fig. 2D–F, spotted bars) elevates the expression levels of all genes compared with the levels seen in control worms (black bars). Yet, while DAF-16 (D) and SKN-1 (F) target genes exhibited average increase levels of 19–37%, the expression level of *hsp-70* was elevated by approximately 170% (E). These results propose that NT219 differentially affects the activity levels of the IIS-regulated transcription factors, displaying a more prominent effect on HSF-1, however; this possibility requires further experimental evaluation. In all cases, the knockdown of *daf-2* by RNAi (gray bars) resulted in larger increase in the expression of the tested genes compared with NT219 treatment (spotted bars). The reduced effect of NT219 on gene expression compared with *daf-2* RNAi was surprising, given the efficient IGF1 signaling attenuation observed in NT219-treated human cells (Fig. 1). Thus, we asked whether the compound’s effect on gene expression emanates from IIS alteration. To test that, we examined whether NT219 modulates the cellular localization of DAF-16. Worms that express GFP-tagged DAF-16 under the regulation of the *daf-16* promoter (strain TJ356) were left untreated (EV), exposed to the vehicle or treated with NT219 for 3 h. An identical group of worms was treated with *daf-2* RNAi. Visualization indicated that unlike in untreated and vehicle-exposed worms, DAF-16 enters the nuclei as a result of NT219 treatment and exposure to *daf-2* RNAi (Fig. 2G, arrows). These observations confirm that NT219 attenuates the IIS.

Thus, the relatively low potency of NT219 compared with *daf-2* RNAi can be possibly explained by differences among the sequences of the nematode’s *daf-2* and the mammalian *Igf1r* (Kimura *et al*., 1997) which result in distinct spatial structures and reduced affinity of NT219 to IIS components. Alternatively, a differential penetrance of the drug to distinct worm tissues may underlie this difference. Further research is required to elucidate this issue.

### Alleviated proteotoxicity in NT219-treated model nematodes

**Figure 3:**
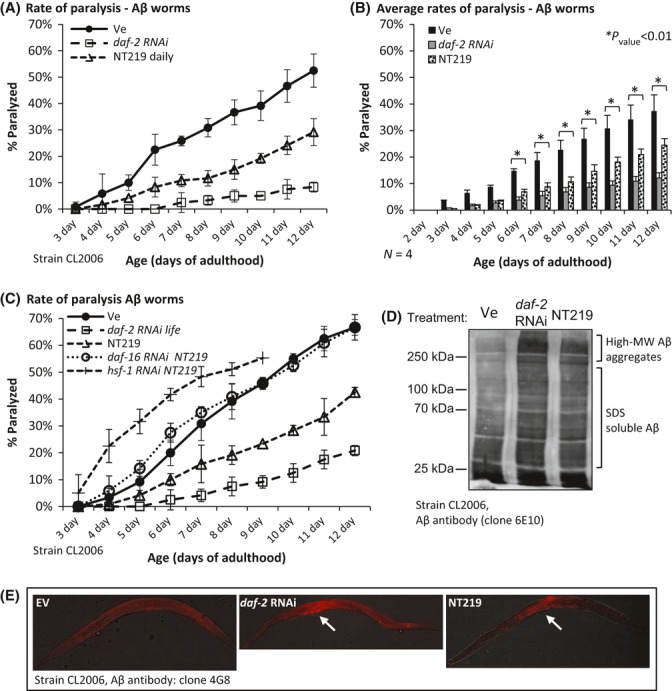
Protection from Aβ-mediated proteotoxicity by NT219. (A) CL2006 worms were cultivated on EV bacteria and treated with either 600 μm NT219 or the vehicle on days 1 and 2 of adulthood. The worms were placed on plates which supplemented daily with NT219 or the vehicle. Rates of paralysis were scored daily from day 3 to day 12 of adulthood. NT219 significantly reduced the rate of paralysis within the worm population (triangles) compared with the control group (black circles). *daf-2* RNAi (open squares) provides more efficient protection compared with NT219. (B) Four independent repeats of the experiment described in A. (C) The knockdown of *daf-16* (open circles) or *hsf-1* (diamonds) by RNAi completely abolished the protective effect of NT219. (D) Similarly to *daf-2* RNAi, NT219 treatment resulted in Aβ hyperaggregation as revealed by WB analysis of high-speed supernatants of CL2006 worms which were treated with the vehicle (Ve), NT219 or with *daf-2* RNAi. (E) Immunofluorescence using an Aβ antibody indicates that IIS reduction by either NT219 or *daf-2* RNAi leads to the accumulation of Aβ in the center of the worm.

The findings that NT219 treatment reduces the activity of the IIS have led us to ask whether this compound can protect worms from Aβ-mediated toxicity. To examine this hypothesis, we employed Aβ worms and the paralysis assay (Cohen *et al*., 2006). The worms were cultivated on EV bacteria and soaked for 3 h at days 1 and 2 of adulthood in either 600 μm NT219 (NT219) or in the vehicle (Ve). From day 3 of adulthood up until termination of the experiment, each worm group was treated daily with the same compound as at days 1 and 2 of adulthood. An additional worm group was treated throughout life with *daf-2* RNAi (*daf-2* RNAi has indistinguishable effects on paralysis of Aβ worms when applied from hatching or from day 1 of adulthood [Fig. S4 and (Cohen *et al*., 2010)]. While 53% of the control worms (Ve) were paralyzed at day 12, daily NT219 treatment reduced the rate of paralysis to merely 29% at the same age. The rate of paralysis within the *daf-2* RNAi-treated animals was 9% (Fig. 3A). Four independent experiments confirmed the significance of this phenomenon (Fig. 3B). To test whether the NT219 counter-proteotoxic effect is conferred by IIS reduction, we asked whether DAF-16 and/or HSF-1 are required for NT219 to protect from paralysis. CL2006 worms were grown from hatching on EV, *daf-16,* or *hsf-1* RNAi bacteria and treated with NT219 or the vehicle as described above. Following the rates of paralysis, we found that the knockdown of either one of these transcription factors abolished the NT219-mediated protection from Aβ aggregation (Fig. 3C). Similarly, we also found that the rates of paralysis within *daf-16* RNAi-treated Aβ worm populations that were either treated with NT219 or exposed to vehicle were nearly identical (Fig. S5). These results indicated that NT219 ameliorates proteotoxicity through IIS inhibition and activation of DAF-16 and HSF-1.

As IIS inhibition protects Aβ worms by hyperaggregating Aβ oligomers (Cohen *et al*., 2006), we tested whether NT219 acts through a similar mechanism. Aβ worms that were grown on control bacteria (EV) were either treated with the vehicle or NT219. A third worm group was grown from hatching until day 2 of adulthood on *daf-2* RNAi bacteria. The worms were homogenized, cleared, and subjected to high-speed centrifugation. Aβ structures in the postdebris supernatants (PDS) were analyzed by WB. Similarly to *daf-2* RNAi, NT219-treated worms contained an elevated amount of high molecular weight Aβ aggregates compared with control animals (Fig. 3D). To test whether NT219 affects Aβ distribution within the worms we used immunofluorescence and found that similarly to *daf-2* RNAi (Cohen *et al*., 2006), treatment with NT219 leads to Aβ accumulation in the center of the worm (Fig. 3E, arrows) supporting the notion that NT219 and *daf-2* RNAi activate the same protective mechanism.

To assess whether NT219 also protects from disease-linked, aggregative proteins other than Aβ, we used worms that express polyQ stretches of either 35 or 40 repeats fused to the yellow fluorescent protein (YFP) in their body wall muscles (strains polyQ35-YFP and polyQ40-YFP, respectively). The expression of either one of the polyQ-YFP constructs leads to the formation of visible foci and to reduced motility in an aging dependent manner. These phenomena can be delayed by IIS reduction (Morley *et al*., 2002).

**Figure 4:**
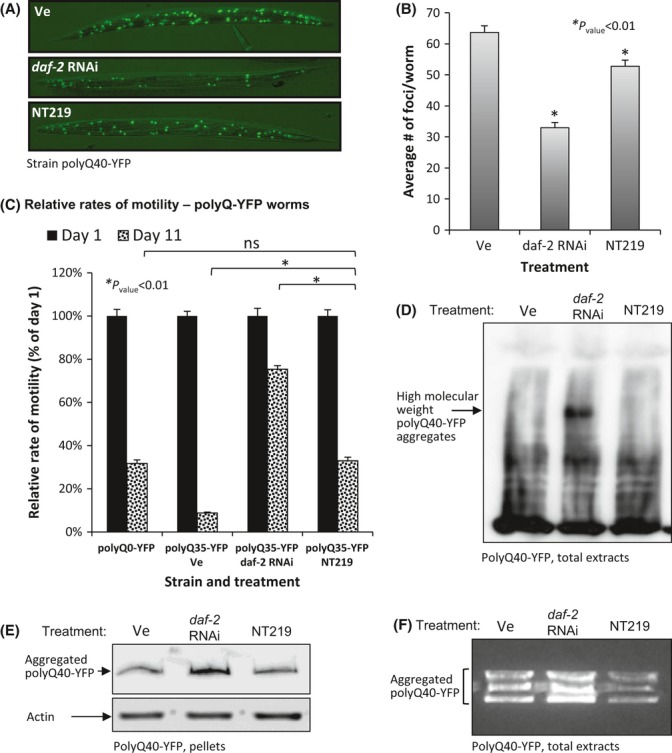
NT219 alleviates polyQ-associated toxicity. (A–B) NT219-treated polyQ40-YFP worms contain significantly fewer polyQ40-YFP foci than their vehicle-treated counterparts as visualized (A) and quantified (B) by fluorescent microscopy (average numbers of foci/worm in vehicle- and NT219-treated animals were 63.66 and 52.78, respectively, *P*_value_ = 0.00026). The effect of NT219 was less prominent than that of *daf-2* RNAi (average of 32.94 foci/worm). (C) NT219 treatment rescues polyQ35-YFP worms from late-life motility impairment. While the average crawling speed of 11-day-old, vehicle-treated polyQ35-YFP worms was 8.8% of the average speed at day 1 of adulthood, the speed of NT219-treated animals was 32.9% of the speed at day 1 (*P*_value_ = 1.21E-29). The average crawling speed of NT219-treated animals was similar to that of day 11 worms that do not express polyQ stretches (polyQ0-YFP, average speed of 31.8% at day 11 compared with day 1 of adulthood). The relative speed of 11-day-old *daf-2* RNAi-treated animals was 75.4% compared with the speed measured at day 1. (D–F) NT219 treatment does not enhance the aggregation rate of polyQ40-YFP as examined by WB of whole worm extracts (D), high-speed pellets of worm debris (E) and by YFP fluorescence assay of worm homogenates separated on native gel (F).

To test whether NT219 affects the number of foci in polyQ40-YFP worms, we counted the fluorescent dots in at least seventy animals of each of the following groups: worms that were grown on EV bacteria and treated with either (i) NT219 or (ii) the vehicle (Ve) at days 1 and 2 of adulthood, as well as in (iii) *daf-2* RNAi-treated worms (Fig. 4A). The average number of foci per worm in the NT219-treated group was significantly lower compared with the animals of the control group (Fig. 4B) but higher than in the *daf-2* RNAi-treated animals.

We also asked whether NT219 mitigates polyQ35-YFP aggregation-mediated motility impairment by measuring the crawling speed of young (day 1) and old (day 11) polyQ35-YFP animals that were cultivated on EV bacteria and treated from day 1 of adulthood either with NT219 or the vehicle (Ve). An identical worm group was grown from hatching on *daf-2* RNAi. As an additional control, we measured the crawling speeds of untreated worms that solely express YFP in their muscles (polyQ0-YFP). Our results (Fig. 4C and Movies S1–S4) show that while the average crawling speed of the vehicle-treated, 11-day-old polyQ35-YFP worms was only 8.8% compared with the speed of 1-day-old animals, their NT219-treated counterparts were largely rescued from polyQ35-YFP-associated motility impairment. Animals of the latter group exhibited crawling speeds similar to those of polyQ0-YFP animals (32.9% and 31.8% respectively), indicating that NT219 largely abolished the toxic effect of polyQ35-YFP aggregation. As seen before, the protective effect of NT219 was less prominent than that of *daf-2* RNAi.

To explore the mechanism by which NT219 protects from polyQ aggregation, we subjected polyQ40-YFP worms to the vehicle, NT219 or *daf-2* RNAi treatments as above, homogenized them at day 2 of adulthood, and analyzed the quantities of SDS-resistant polyQ40-YFP aggregates by WB. Highly aggregated molecules were apparent in homogenates of *daf-2* RNAi-treated worms, but not in homogenates of vehicle- or NT219-treated animals (Fig. 4D). Analogous results were obtained when homogenates were prepared as above and supernatants were separated from pellets by high-speed centrifugation and analyzed by WB. While treatment with *daf-2* RNAi resulted in increased amounts of aggregated polyQ40-YFP compared with vehicle-treated animals (Ve), no increase was conferred by NT219 (Fig. 4E).

We also analyzed the amounts of polyQ40-YFP aggregates using native gels. PolyQ40-YFP animals were grown and treated with either the vehicle (Ve), NT219 or *daf-2* RNAi, homogenized, and loaded onto a 1% native gel that preserves polyQ40-YFP aggregates and enables YFP to fluoresce (van Ham *et al*., 2010). YFP fluorescence levels were compared. Our results (Fig. 4F) showed that contrarily to *daf-2* RNAi treatment, NT219-treated worms did not exhibit elevated rates of polyQ40-YFP aggregation as indicated by the low fluorescence levels compared with the control animals.

Our observations propose that although both *daf-2* RNAi and NT219 protect worms from polyQ40 aggregation-mediated toxicity, these treatments confer protection by differentially modulating the disaggregation and hyperaggregation mechanisms of the worm.

### Elevated resistance to heat and to ultraviolet radiation exhibited by NT219-treated worms

**Figure 5:**
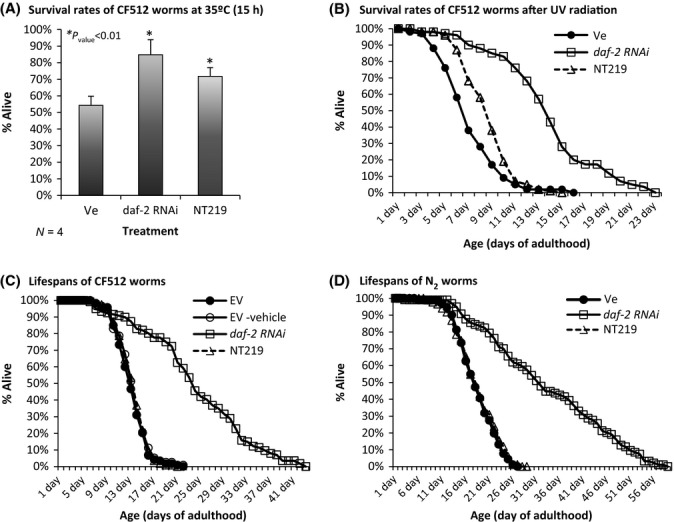
NT219 elevates stress resistance but has no effect on lifespan. (A) NT219-treated CF512 worms exhibit significantly elevated survival rate (71.7%) after 15 h of exposure to 35°C compared with their vehicle-treated counterparts (54.3%, *P*_value_ = 0.008) but lower than *daf-2* RNAi-treated animals (84.8%, *P*_value_ = 0.0001). (B) CF512 worms were treated as in A, exposed to sublethal UV dose, and their survival rates were recorded daily. NT219-treated animals lived longer than vehicle-treated worms (8.76 ± 0.21 and 7.03 ± 0.22 days, respectively, *P*_value_ = 2.36E-8) but less than their *daf-2* RNAi-treated counterparts (13.89 ± 0.41 days). (C) Untreated worms (black circles), NT219-treated animals (triangles), and their counterparts that were supplemented with the chemical vehicle (open circles) exhibit indistinguishable lifespans. *daf-2* RNAi-treated worms were long-lived (squares). (D) The lifespans of wild-type worms (strain N2) were not affected by a daily NT219 treatment.

The elevated expression of protective genes in NT219-treated worms has led us to ask whether this compound elevates the worms’ resistance to environmental insults. First, we tested whether NT219 elevates resistance to heat. CF512 worms that were grown on EV bacteria to day 1 of adulthood were divided into two identical groups. One group was treated with the vehicle (Ve), while the other was treated with NT219 for 3 h at days 1 and 2 of adulthood. A third group was treated with *daf-2* RNAi from hatching to harvest. The worms were exposed to 35°C, and rates of survival were scored after 15 h. Results of four independent experiments (Fig. 5A) indicated that NT219 raised the survival rates within the worm populations by 33% compared with the control group (survival rates of 72% and 54%, respectively). *daf-2* RNAi-treated worms exhibited an average survival rate of 85%.

Next, we tested whether NT219 elevates resistance to DNA damage by ultraviolet (UV) radiation, a feature of IIS reduction (Murakami & Johnson, 1996). Two groups of CF512 worms were grown on EV bacteria. At day 1 of adulthood, the worms were treated with either the vehicle (Ve) or NT219 and exposed to sublethal UV dose. The worm populations were supplemented daily with the vehicle or NT219, and their survival rates were recorded. A third group of worms was grown on *daf-2* RNAi bacteria. NT219 significantly elevated the survival rate of UV-radiated worms (Fig. 5B, mean survival rates (days ± SEM): vehicle-treated control 7.03 ± 0.22, NT219-treated 8.76 ± 0.21 *P*_value_ = 2.36E-8). As observed for heat stress, the effect of NT219 on the survival of UV-radiated animals was less prominent than that of *daf-2* RNAi (average survival 13.89 ± 0.41 days).

### NT219 has no effect on the nematode’s lifespan

As longevity is a hallmark of IIS reduction (Kenyon *et al*., 1993), we tested whether NT219 influences the worms’ lifespan. CF512 worms were treated with NT219 in two regimes; (i) EV bacteria-grown worms were soaked for 3 h in either NT219 or the vehicle at days 1 and 2 of adulthood. The worms were then transferred onto plates spotted with EV bacteria and supplemented daily with either the vehicle or the drug. A third group was fed *daf-2* RNAi bacteria throughout life, and a fourth group was grown on EV bacteria throughout development and adulthood. (ii) As IIS reduction regulates lifespan solely during reproductive adulthood (Dillin *et al*., 2002), we performed a second experiment in which we maximized the worms’ exposure to the drug during this period of life. EV bacteria-grown CF512 worms were treated daily with either NT219 or the vehicle during days 1–6 of adulthood. At day 6, the worms were transferred onto plates seeded with EV bacteria and lifespans were recorded. Worms that were treated with *daf-2* RNAi throughout life served as a control group. In both setups, NT219-treated and untreated worm populations exhibited indistinguishable lifespans, indicating that NT219 has no effect on lifespan (Figs 5C and S6). Similarly, the lifespans of wild-type (strain N2) worm populations that were either treated daily with NT219 or left untreated were identical (Fig. 5D).

## Discussion

The therapeutic potential of IIS inhibition as a treatment for neurodegenerative maladies has been demonstrated by a plethora of studies. Here, we assessed whether, the novel IIS inhibitor, NT219 exhibits counter-proteotoxic activity and can potentially serve as a drug to treat neurodegenerative disorders. Employing model nematodes, we found that NT219 mitigates the toxic effects of neurodegeneration-linked protein aggregation. Our mechanistic investigation unveiled that NT219 efficiently blocks IGF1R to AKT signaling in mammalian cells by a dual-step mechanism. It reduces the autophosphorylation of the IGF1R to prevent the IGF1-induced phosphorylation and activation of AKT and induces a negative feedback loop, directing IRS1/2 for inhibitory Ser-phosphorylation and degradation. NT219 was also found to increase the expression levels of FOXO target genes in mammalian cells and downstream of all three known IIS-regulated transcription factors of *C. elegans*, DAF-16, HSF-1, and SKN-1 and to elevate the nematode’s resistance to heat and UV radiation. Surprisingly, unlike *daf-2* RNAi treatment, NT219 has no effect on lifespan.

These findings raise the question of how NT219 differentially affects the distinct functions downstream of the IIS, protecting from proteotoxicity and elevating stress resistance but having no influence on lifespan. A few models can explain this phenomenon. Perhaps the most plausible explanation relies on the differential transcriptional effect of the drug on the gene networks regulated by the different transcription factors. This model proposes that elevated levels of chaperones can increase stress resistance and protect from proteotoxicity but is not sufficient to increase lifespan. This theme is supported by several studies which indicate that longevity, proteostasis, and stress resistance are separable. First, it was reported that worms that lack the genes that encode all five superoxide dismutase (*sod*) genes, which are critically required for oxidative stress resistance, exhibit normal lifespan (Van Raamsdonk & Hekimi, 2012). Intriguingly, the knockdown of *sod-2* resulted in extended lifespan (Van Raamsdonk & Hekimi, 2009), indicating that stress resistance and extended lifespan may be inversely related. Recently, we found that reducing heat stress resistance by knocking down *gtr-1*, a gene that encodes a neuronal receptor, has no effect on lifespan (Maman *et al*., 2013). In addition, the longevity and counter-proteotoxic functions of the IIS are temporally separable as late-life IIS reduction protects worms from Aβ toxicity without affecting lifespan (Cohen *et al*., 2010). Together, these studies suggest that different mechanisms underlie the distinct effects of IIS reduction.

On the other hand, the maintenance of proper protein homeostasis (proteostasis) and lifespan has been shown to be tightly linked. Elevated expression of chaperone-encoding genes protects worms from proteotoxicity (Prahlad & Morimoto, 2011) and is required for the full longevity phenotype of IIS mutants (Murphy *et al*., 2003; Morley & Morimoto, 2004). In addition, compounds that bind protein aggregates and protect from proteotoxicity increase lifespan (Alavez *et al*., 2011). Yet, although it is possible that efficient proteostasis is a prerequisite for longevity, it may not be sufficient to extend lifespan if other conditions were not fulfilled. According to this model, NT219 activates the mechanisms that protect from proteotoxicity but fails to meet the complete set of requirements to extend lifespan. While it is unclear what the requirements for longevity are, it is likely that proper lipid metabolism and robust genome maintenance are among these conditions.

A second model suggests that NT219 does not extend lifespan due to the relative moderate induction of IIS target genes compared with *daf-2* RNAi. According to this view, the enhanced expression of IIS target genes has to reach a threshold level to affect lifespan. NT219 fails to reach this threshold level, and thus, although it partially protects from proteotoxicity and moderately elevates stress resistance, these modulations are not sufficient to prolong life.

A third model relies on the observation that the IIS confers different functions in distinct tissues. While DAF-16 is expressed in several tissues, it plays its longevity roles mainly in the intestine (Libina *et al*., 2003). Recently, it was reported that lifespan modulation by DAF-16 is also depends on intertissue communication (Zhang *et al*., 2013). Accordingly, it is possible that NT219 differentially penetrates distinct tissues and acts in some cell types but not in others.

Although the different effects of the compound on the expression levels of genes downstream of DAF-16 and HSF-1 suggest that the first model is more likely, the high competence of NT219 as an IGF1 signaling inhibitor in mammalian cells (Fig. 1) implies that it may be able to extend lifespan of mammals. Further research is required to decipher how NT219 differentially affects IIS functions.

Surprisingly, we found that while *daf-2* RNAi treatment elevates the rates of aggregation of both Aβ and polyQ40-YFP, NT219 enhances Aβ aggregation, but not the rate of polyQ40-YFP aggregation. The opposing effects of NT219 on these aggregative peptides are puzzling as the compound protects both Aβ and polyQ35-YFP worms from proteotoxicity. This can be explained by the opposing activities that are regulated by the IIS, disaggregation, and hyperaggregation. Disaggregation appears to be the preferred detoxification mechanism while hyperaggregation serves as a secondary line of defense (Cohen *et al*., 2006). According to this model, the knockdown of *daf-2* by RNAi strongly induces the activities of both protective mechanisms resulting in net elevated amounts of Aβ and polyQ40-YFP aggregates. As the inhibition of the IIS by NT219 is less prominent than that of *daf-2* RNAi, it is plausible that when an elevated aggregation challenge is presented by Aβ, the different treatments activate both detoxification mechanisms. However, when the worms face a less challenging insult of polyQ40-YFP aggregation, NT219 only activates the disaggregation mechanism and a reduction in the total amount of polyQ40-YFP aggregates is observed.

The indications that NT219 protects worms from the toxicity of two neurodegeneration-linked aggregative peptides, Aβ and polyQ35-YFP, show that this compound has the potential to combat more than one neurodegenerative disorder. This is one of the most attractive aspects of the aging manipulation as a counter-neurodegeneration strategy, having one treatment for different maladies. The lack of an effect on lifespan promises to inhibit the progression of neurodegeneration rather than postpone it to later stages of life.

Several key questions have to be addressed to further scrutinize the therapeutic potential of NT219. First, it is required to test whether this drug can mitigate proteotoxicity in AD and HD model mice. The high potency of the compound seen in mammalian cells suggests that NT219 will exhibit an increased therapeutic competence in the mouse. It is also required to develop efficient administration route.

In conclusion, this study supports the notion that longevity and protection from aging-associated diseases are separable and strengthens the theme that selective aging manipulations by small molecules that inhibit the IIS bear the promise to delay the emergence and slow the progression of late-onset neurodegenerative maladies.

## Experimental procedures

### Worm and RNAi strains

All worm strains were obtained from the Caenorhabditis Genetics Center (CGC, Minneapolis, MN, USA). The worms were grown at 20°C. CF512 [fer-15(b26)II; fem-1(hc17)IV] was grown at 15°C. To avoid progeny, eggs of CF512 worms were incubated at 20°C for 16 h, larvae transferred to 25°C for 48 h and back to 20°C. To reduce gene expression, we used previously described (Dillin *et al*., 2002) bacterial strains expressing dsRNA: empty vector (pAD12), *daf-16* (pAD43), and *daf-2* (pAD48). RNAi toward *hsf-1* was from the Ahringer library. RNAi bacteria were grown at 37°C in LB medium with 100 μg mL^−1^ ampicillin and seeded onto NG-ampicillin plates supplemented with 100 mm Isopropyl β-D-1-thiogalactopyranoside (IPTG).

### Protein blotting and analysis

Human melanoma A375 cells were obtained from the ATCC and cultured in RPMI with 10% FCS and antibiotics. The cells were cultured in a serum-starved medium, stimulated with 50 ng mL^−1^ IGF1 for 5 min, and lysed with boiling buffer (10% glycerol, 50 mm Tris–HCl, pH6.8, 3% SDS, and 5% 2-mercaptoethanol). Equal amounts of protein per sample were subjected to SDS-PAGE and immunoblotted with anti-p(T308) PKB (cat#9275), anti-p(S473) PKB (cat#9271), anti-p(Y1131) IGF1Rβ/(Y1146) IR (cat#3021) and anti-pS(636/639)IRS1 (cat#2388) antibodies (Cell Signaling Technology, Beverly, MA, USA), and anti-p(Y612)IRS1 (cat#44-816G) antibody (Biosource, Grand Island, NY, USA), to detect the phosphorylation levels (‘p’ in figures) of the indicated proteins. Following stripping, the membranes were immunoblotted with anti-IRS1 (cat#ab40777) and anti-IRS2 (cat#ab52606) antibodies (Abcam, Cambridge, UK), anti-PKB1/2 (cat#sc8312) and anti-IGF1Rβ (cat#sc731) antibodies (Santa Cruz Biotechnology, Santa Cruz, CA, USA) and anti-β the membranes were immunoblotted with anti-IRS1 (cat#ab40777) and anti-IRS2 (cat#ab52606) antibodies [Abcam (Camb-react with the corresponding phosphorylated forms of IRS2 and therefore marked as pS-IRS1/2 and pY-IRS1/2. Antibodies toward p27 (cat# sc-528; Santa Cruz Biotechnology) and toward Mn-SOD (cat#06-984; Millipore, Billerica, MA, USA) were used to follow the expression levels of these proteins.

Worms were grown on RNAi bacteria and treated with NT219 as indicated. NT219 was diluted in 20% 2-hydroxypropyl-β-cyclodextrin (2-HP-β-CD) (Sigma, St. Louis, MO, USA) and diluted to the indicated concentration in the buffer reaction. The vehicle, 20% 2-HP- β-CD, was diluted identically in the control systems. The worms were resuspended in cold PBS and homogenized in a tissue grinder (885482, Kontes, Vineland, NJ, USA). Homogenates were spun (3000 rpm, 3 min) at 4°C. Total protein concentrations in the supernatants were measured using Bradford reagent (#500-0006; Bio-Rad, Hercules, CA, USA). Proteins were separated on gel, transferred onto a PVDF membrane and blotted by anti-GFP antibody (cat #2956; Cell Signaling, Danvers, MA, USA) or Aβ antibody (SIG-39320; Covance, Emeryville, CA, USA). Chemiluminescence was detected using an Image Analyzer (LASS-3000- Fujifilm, Tokyo, Japan). For fluorescent detection of polyQ40-YFP aggregates, native loading dye (2×: 0.125 m Tris pH6.8, 20% Glycerol, 0.02% Bromophenolblue) was added to an equal amount of worm homogenates, and the samples were loaded onto a 1% native agarose gel, dissolved in running buffer (25 mm Tris pH8.3, 0.19 m Glycine), and run at 50 V for 20 h. Images were taken using the LASS-3000 system, and band intensities were measured using ImageJ software. Statistical significance was calculated using the *t*-test function of the Excel software.

## Supporting Information

**Fig. S1** NT219 elevates the levels MnSOD in Neuroblastoma 2a (N2a) cells. **Fig. S2** NT219 elevates the activity of the *hsp-16.2* promoter. **Fig. S3** BMS-754807 has no effect on the activity of the *hsp-16.2* promoter. **Fig. S4** *daf-2* RNAi treatment has indistinguishable counter-proteotoxic effects when applied from hatching or from day 1 of adulthood. **Fig. S5** NT219 has no effect on paralysis of *daf-16* RNAi-treated Aβ worms. **Fig. S6** NT219 treatment during reproductive adulthood does not affect lifespan. **Table S1** Relative motility of polyQ35-YFP worms (corresponding Figure 4C). **Table S2** Lifespans (corresponding Figures 6C,D).

**Movie S1** Day 11 old polyQ0-YFP worms – untreated.

**Movie S2** Day 11 old polyQ35-YFP worms – vehicle-treated.

**Movie S3** Day 11 old polyQ35-YFP worms – NT219 treated.

**Movie S4** Day 11 old polyQ35-YFP worms – *daf-2* RNAi treated.

**Data S1** Experimental procedures.
