# Supplementary material for: A novel inhibitor of the insulin/IGF signaling pathway protects from age-onset, neurodegeneration-linked proteotoxicity
Source: Aging Cell. 2013 Nov 22;13(1):165–74. doi: 10.1111/acel.12171 (PMC4326862; doi:10.1111/acel.12171)
Supplement: Supplementary file 1 — Fig. S1 NT219 elevates the levels MnSOD in Neuroblastoma 2a (N2a) cells. Fig. S2 NT219 elevates the activity of the hsp-16.2 promoter. Fig. S3 BMS-754807 has no effect on the activity of the hsp-16.2 promoter. Fig. S4 daf-2 RNAi treatment has indistinguishable counter-proteotoxic effects when applied from hatching or from day 1 of adulthood. Fig. S5 NT219 has no effect on paralysis of daf-16 RNAi-treated Aβ worms. Fig. S6 NT219 treatment during reproductive adulthood does not affect lifespan. Table S1 Relative motility of polyQ35-YFP worms (corresponding Figure 4C). Table S2 Lifespans (corresponding Figures 6C,D). [file acel0013-0165-sd1.pdf]

Supplementary figures

Figure S1

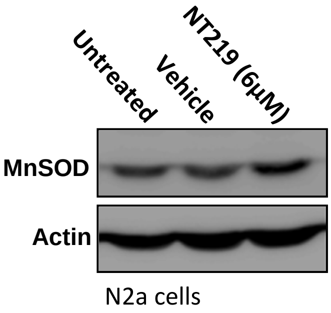

Figure S2

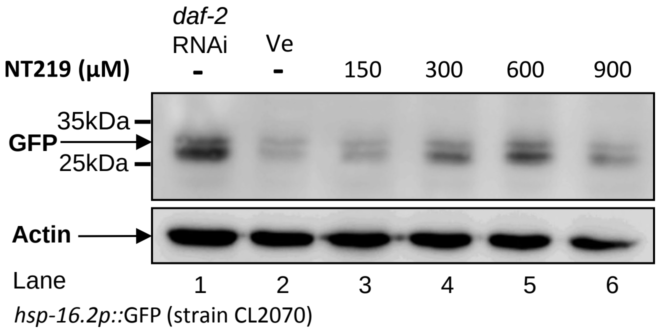

Figure S3

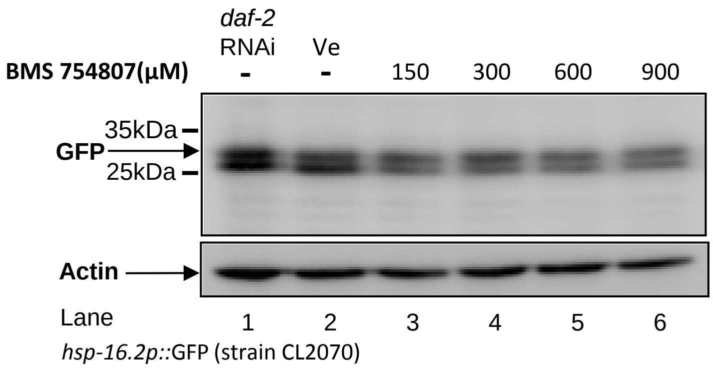

Figure S4

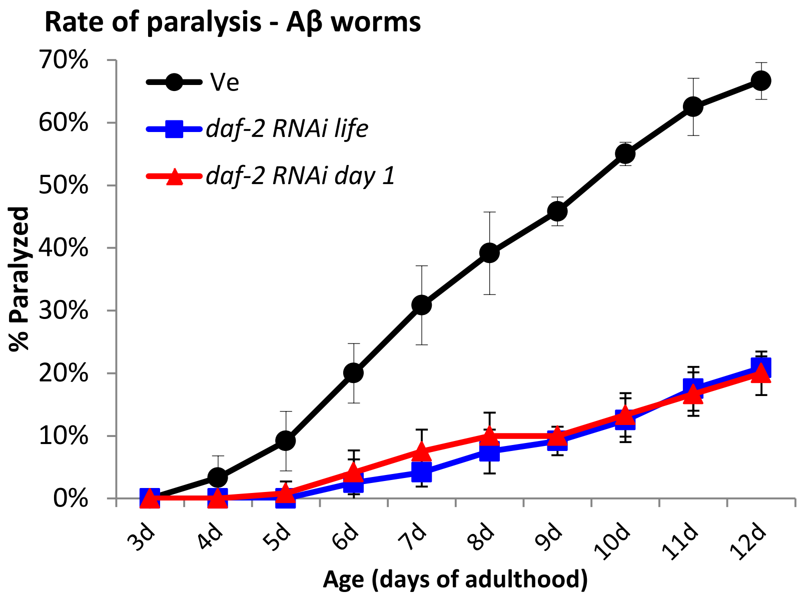

Figure S5

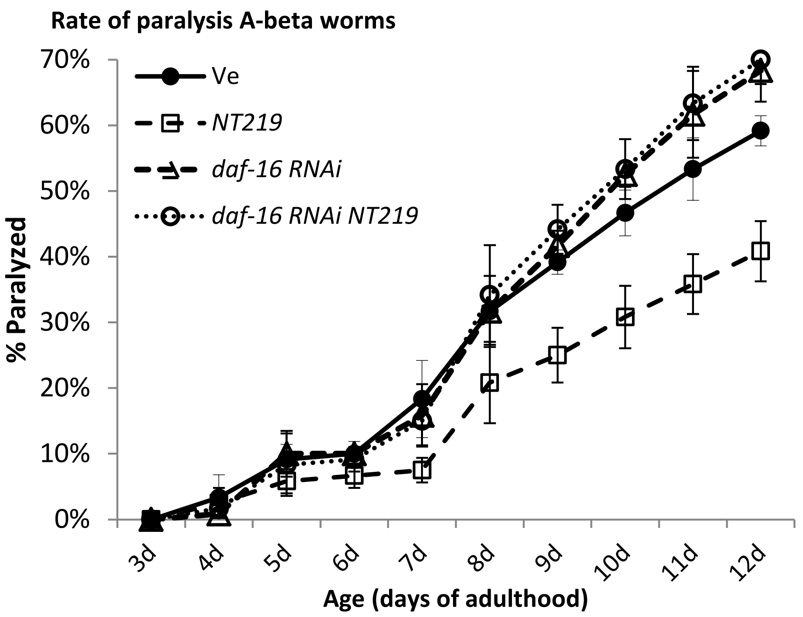

Figure S6

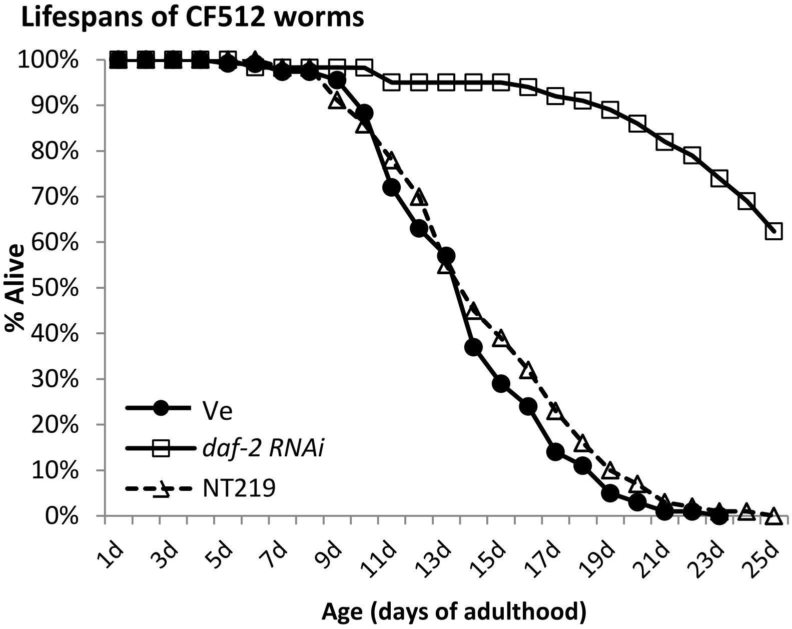
Lifespans of CF512 worms
100%
90%
80%
70%
60%
50%
40%
30%
20%
10%
0%
% Alive
Ve
daf-2 RNAi
NT219
1d
3d
5d
7d
9d
11d
13d
15d
17d
19d
21d
23d
25d
Age (days of adulthood)

Supplementary table 1

Relative motility of polyQ35-YFP worms (corresponding figure 4C)

Strain	Treatment	n	Average speed (µm/sec) day 1	Average speed (µm/sec) day 11	Relative speed day 1/day 11	*p* value (day 11) vs. polyQ0-YFP (EV)	vs. polyQ35-YFP (EV)	vs. polyQ35-YFP (*daf-2* RNAi)
polyQ0-YFP	**None (EV)**	56	46.4 ± 1.41	14.74 ± 0.75	31.8%			
polyQ35-YFP	**None (EV)**	56	69.65 ± 2.07	6.14 ± 0.38	8.8%	<0.0001		
polyQ35-YFP	***daf-2* RNAi**	56	54.0 ± 1.93	40.7 ± 0.89	75.4%	<0.0001	<0.0001	
polyQ35-YFP	**NT219**	56	51.21 ± 1.49	16.87 ± 0.84	32.9%	>0.05	<0.0001	<0.0001

Supplementary table 2

Lifespans (corresponding figures 6C, D)

Strain	Treatment	Mean lifespan ± SEM (days)	number of animals / initial number	% lifespan of control	P value (against control)
CF512	**EV**	13.28 ± 0.24	119 / 120		
	**EV-Vel**	13.46 ± 0.26	116 / 120	101	0.61
	**EV-NT219**	13.47 ± 0.24	114 / 120	101	0.56
	***daf-2* RNAi**	24.07 ± 0.83	114 / 120	181	2.62E-28
N2	**Ve**	18.42 ± 0.44	91 / 109		
	**EV-NT219**	18.45 ± 0.49	83 / 108	100	0.43
	***daf-2* RNAi**	33.35 ± 1.32	93 / 113	181	8.87E-21
