# Supplementary material for: A novel inhibitor of the insulin/IGF signaling pathway protects from age-onset, neurodegeneration-linked proteotoxicity
Source: Aging Cell. 2013 Nov 22;13(1):165–74. doi: 10.1111/acel.12171 (PMC4326862; doi:10.1111/acel.12171)
Supplement: Supplementary file 6 — Data S1 Experimental procedures. [file acel0013-0165-sd6.docx]

**Data S1 Experimental procedures**

***RNA isolation and Quantitative Real-Time PCR***

Total RNA was isolated from synchronized populations of approximately 15,000 sterile worms grown at 20ºC for the indicated age using QIAzol reagent (Cat #79306 QIAGEN, Hilden Germany) and purified by RNeasy lipid tissue kit (QIAGEN #74104). cDNA was created using iScript advanced cDNA Synthesis Kit (BIO-RAD, #170-8891). For quantitative PCR reactions, dilutions of 1:10 were used. Real-time qPCR experiments were performed as described in the manual using CFX cycler (Bio-Rad, Hercules, CA USA) and EvaGreen supermix (Bio-Rad, #172-5204). Quantification was normalized to control levels of *act-1* cDNA.

*sod-3* primer set: Forward: CTA AGG ATG GTG GAG AAC CTT CA
Reverse: CGC GCT TAA TAG TGT CCA TCA G

*hsp-12.6* primer set: Forward: TTCCAGTGATGGCTGACG; Reverse: GGCTTCTAGGCCTACTTCG

*hsp-70* primer set: Forward: GGTTGGGGGATCAACTCG
Reverse: CACCAAAGGCTACTGCTTCG.

*hsp-16.1:* Forward: Reverse:
*gst-4:* Forward: CCCATTTTACAAGTCGATGG
Reverse: CTTCCTCTGCAGTTTTTCCA
*gst-10*: Forward: GTCTACCACGTTTTGGATGC
Reverse: ACTTTGTCGGCCTTTCTCTT
*act-1* primer set: Forward: GAGCACGGTATCGTCACCAA
Reverse: TGTGATGCCAGATCTTCTCCAT.

***Worm immunofluorescence***

Immunofluorescence was performed as described previously ([Cohen et al, 2006](#_ENREF_10)). Briefly, worms were prefixed with 4% paraformaldehyde in MRWB (80mM KCl, 20mM NaCl, 10mM EGTA, 5mM Spermidine, 50% Methanol), froze on dry ice, incubated on ice for 1h and washed once in M9 buffer and twice more in Tris–Triton buffer (100mM Tris pH7.4, 1% TX-100,1mM EDTA). The worms were then incubated for 2h at 37°C in Tris–Triton buffer supplemented with 1% β-Mercaptoethanol, washed in BO_3_ buffer (25mM H_3_BO_3_, 12.5mM NaOH) and incubated for 15min in BO_3_ buffer supplemented with 10mM DTT. Next, the worms were washed and incubated in BO_3_ buffer supplemented with 0.3% H_2_O_2_ for 15min (RT) and washed with BO_3_ buffer and with blocking buffer (PBSX1, 1% BSA, 0.5% TX-100, 1mM EDTA). Aβ staining was performed overnight at 4°C using 4G8 antibody, washed in blocking buffer and stained for 30min (RT) with a secondary antibody conjugated to rhodamin.

***Lifespan analysis***

Synchronized worm eggs were placed on master NG-ampicillin plates seeded with the indicated RNAi bacterial strain and supplemented with 100mM IPTG. The eggs were incubated at 20°C until transferred onto small NG-ampicillin plates (10 animals per plate) at the indicated ages. Adult worms were transferred onto freshly seeded plates every four days. Worms that failed to move their noses when tapped twice with a platinum wire were scored as dead. Dead worms were scored daily. Lifespan analyses were conducted at 20°C.

***Heat and UV stress assays***

Synchronous eggs of CF512 worms were placed on NG plates containing 100μg/mL ampicillin, seeded with the indicated bacterial strain (EV or *daf-2* RNAi) and supplemented with 100mM IPTG. The worms were incubated at 20°C until day 1 of adulthood and treated as indicated. Total of 120 worms were transferred onto fresh plates spotted with bacteria. The plates were exposed to 35˚C for 15 hours and vitality rates were recorded. Worms that did not respond to tapping were scored dead. To evaluate resistance to UV radiation, eggs of CF512 worms were placed on plates and treated as described above. Day 1 adult, EV-grown worms were treated with NT219 or with the vehicle for 3 hours and exposed to 800j/cm^2^ UV. The worms were transferred onto fresh plates and NT219 or the vehicle was added to the plates daily after scoring viability.

***Paralysis and motility assay*s**

Synchronous CL2006 worm populations were grown on NG plates containing 100µg/ml ampicillin and spotted with EV or *daf-2* RNAi bacteria. At days 1 and 2 of adulthood the worms were either treated for three hours with 600μM NT219 or with the chemical vehicle in solution. 120 worms were placed on 10 plates (12 animals per plate) and the plates were divided randomly to 5 sets (2 plates, 24 worms per set). NT219 or the chemical vehicle was supplemented daily to the plates after testing the rate of paralysis by tapping their noses with a platinum wire. Worms that moved their noses but failed to move their bodies were scored as ‘paralyzed’ and removed from the plates. Paralysis assays were terminated at day 12 of adulthood.
 To test the rates of motility, polyQ35-YFP worms were treated daily with either the chemical vehicle or with 600μM NT219 from day 1 to day 11 of adulthood. As control groups we used polyQ35-YFP that were grown on *daf-2* RNAi bacteria and polyQ0-YFP animals that were grown on EV bacteria and supplemented daily with the chemical vehicle. At days 1 and 11 of adulthood 56 worms of each treatment were transferred onto 8 plates (7 animals/plate) and video-taped by an automated microscope system (30 frames per minute for 1 minute). Crawling speeds were calculated by a worm tracking software.
